# Preparation and Application of Core–Shell Nanocarbon-Based Slow-Release Foliar Fertilizer

**DOI:** 10.3390/nano15070565

**Published:** 2025-04-07

**Authors:** Ting Zhang, Xinheng Chen, Hongtao Gu, Huayi Chen, Kaichun Huang, Jinjin Wang, Huijuan Xu, Yulong Zhang, Wenyan Li

**Affiliations:** 1 Guangdong Province Key Laboratory for Agricultural Resources Utilization, College of Natural Resources and Environment, South China Agricultural University, Guangzhou 510642, China; zt199812052025@163.com (T.Z.); xhchen1999@163.com (X.C.); 17633974630@163.com (H.G.); 15863418988@163.com (K.H.); wangjinjin@scau.edu.cn (J.W.); hjxu@scau.edu.cn (H.X.); 2 Key Laboratory of Arable Land Conservation (South China), MOA, College of Natural Resources and Environment, South China Agricultural University, Guangzhou 510642, China; 3 Guangdong Research Center for Agricultural Soil Pollution Prevention and Control Engineering Technology, College of Natural Resources and Environment, South China Agricultural University, Guangzhou 510642, China; 4 School of Tropical Agriculture and Forestry, Hainan University, Haikou 570228, China; huayi93@hainanu.edu.cn

**Keywords:** nanomaterials, core–shell structure, foliar application, sustained-release performance, *Brassica rapa*

## Abstract

The application of nanotechnology offers a promising solution to improve fertilizer utilization efficiency by mitigating the losses and volatilization of conventional fertilizers, contributing to sustainable agriculture. In this study, a core–shell nanocarbon-based slow-release foliar fertilizer (CN@mSiO_2_-NH_2_@Urea@PDA) was synthesized using nanocarbon (CN) as the core, amino-functionalized mesoporous silica (mSiO_2_-NH_2_) as the shell, and polydopamine (PDA) as the coating layer. BET analysis revealed a 3.5-fold and 1.9-fold reduction in material porosity after PDA encapsulation, confirming successful synthesis. The controlled-release performance was enhanced, with a 24% decrease in the release rate and a prolonged nutrient delivery duration. Hydrophobicity tests demonstrated a 20° increase in the contact angle, indicating improved adhesion. Seed germination assays validated biosafety, while field trials showed a 69.94% increase in the choy sum (*Brassica rapa*) yield, 21.64% higher nitrogen utilization efficiency, and 22.21% reduced nitrogen loss. The foliar application increased the plant nitrogen use efficiency by 18.37%. These findings highlight the potential of CN@mSiO_2_-NH_2_@Urea@PDA as an advanced foliar fertilizer, providing a strategic approach to promote nanomaterial applications in agriculture and enhance the acceptance of functional fertilizers among farmers.

## 1. Introduction

The population explosion worldwide has led to rapidly increased food demands [1]. Fertilizer is important in improving the yield and quality of crops, fruits, vegetables and oil plants. Still, a large number of studies have shown that the nutrient loss caused by the irrational application of fertilizer has become one of the most important sources of agricultural non-point source pollution at present [2,3]. The utilization rate of fertilizer can be improved and nutrient loss can be reduced by the use of slow controlled-release fertilizer. However, the residue from the current commercial slow controlled-release fertilizer is difficult to degrade, leading to the potential for causing other forms of pollution [4]. Root fertilization has a lower usage rate than foliar fertilization [5,6]. However, the crop leaf surface, which has great hydrophobicity along with poor adhesion owing to the lotus leaf effect, caused the loss of leaf surface fertilizer [7]. The escape of foliar fertilizer into soil and other media by downpours flushing and irrigation creates substantial environmental damage and restricts the future application of foliar fertilizer [8].

Nanomaterials, renowned for their diminutive size and significant surface area-to-volume ratio, were utilized to enhance the efficacy and precision of fertilizers [9,10]. Furthermore, as a unique transporter of agrochemicals, it enables targeted and controlled nutrient delivery while considerably enhancing crop yields [11,12]. Although a single component of nanomaterials shows excellent performance, the current classical nanomaterials have been unable to meet the multiple needs. It was an effective means to optimize the properties of the core–shell structure by combining nanotechnology with the core–shell structure and designing and micro-controlling its composite materials [13]. Nanocarbon materials were often used as plant growth enhancers and fertilizer enhancers in agricultural production because of their good biocompatibility [10,14]. At present, many preparation methods of nanocarbon have been developed in plasma treatment, chemical vapor deposition [15], laser evaporation [16], chemical stripping [17], microwave-assisted technology [18], and so on. Among them, the hydrothermal method was an ideal method for preparing nanocarbon due to its advantages of simple operation, a wide range of carbon sources, economic and environmental protection, simple equipment, and rich functional groups on the surface of synthetic products [19].

In recent years, the synthesis of inorganic nanocomposites has attracted extensive attention due to its wide application in industrial and biological fields [20,21]. Inorganic nanocomposites have been used in controlled-release applications due to their non-toxic, biocompatible, and stable properties [22]. Among them, oxidized silica composed of silicon elements was the most prominent carrier or matrix in inorganic nanocomposites [23]. Mesoporous silicon dioxide (MSN) with a hollow channel honeycomb structure was a commonly used carrier of silicon-based nanomaterials [24]. Although a single component of nano-silicon materials shows an excellent performance, it has been unable to meet the needs of multiple applications in the market at this stage. For example, mesoporous silicon can be used as a sustained-release carrier of pesticides to increase the utilization rate of pesticides. However, due to its poor adhesion, it was difficult to attach to the surface of the leaf [25]. Core–shell silicon matrix composite nanoparticles with a special hierarchy structure can modify and cut the core–shell structure to regulate many properties of composite materials, and have a great potential application value in chemical assembly [26], materials science [27], drug delivery [28], agriculture [29], and other fields.

Physical or chemical methods to make nutrient-rich ores or soil-insoluble elements directly into nanoscale fertilizers, or the use of nanotechnology to make fertilizers directly into nanoscale fertilizers can be called nanostructured fertilizers [30]. Nanostructured fertilizers can stimulate plant growth and increase crop yields [31]. In terms of fertilizer release, mesoporous silica core–shell nanoparticles were easy to create effective delivery carriers and packaging materials because their morphology, intermediate structure, and porosity were controllable, and they have easily modified inner and outer surfaces and high biocompatibility [10,32]. Polydopamine (PDA), as a renewable, natural, non-toxic, biodegradable biopolymer, was rich in catechol and amino groups, which were the same as the viscous foot proteins Mefp-3 and Mefp-5 secreted by mussels in nature, and has strong adhesion properties. It was widely used in the surface modification of nanocarriers to further expand the properties of nanocomposites [33,34]. Xiang et al. prepared a composite slow-release fertilizer with an improved slow-release effect using silica nanoparticles as the carrier material [35]. Ji et al. prepared a pesticide–fertilizer nanocomposite based on positively charged functionalized mesoporous silica and polydopamine, and confirmed that polydopamine can improve the adhesion properties of the composite as a foliar fertilizer and reduce losses [36]. The preceding research demonstrates the viability of using silicon-based composite nanoparticles in the field of mitigating fertilizer. Using nanocarbon as a fertilizer synergist can improve the fertilizer utilization rate, reduce nutrient loss, and stimulate plant growth. Additionally, it helps mitigate environmental pollution from volatilization and leaching, which are common issues due to the low efficiency of conventional fertilizers [14].

This study aims to develop a slow-release foliar fertilizer based on nanocarbon with a core–shell structure, evaluating its adhesion, release efficiency, and impact on plant growth. In this study, nanocarbon was used as the core, mesoporous silica as the shell, the two composite materials were amino-modified, and polydopamine was used to block the mesoporous to prepare a slow-release and foliar application of the dual function of the nano slow-release fertilizer. Its nutrient release performance and mechanism as a slow-release fertilizer were explored through the release kinetics test, its actual adhesion performance as a foliar fertilizer was explored through the adhesion evaluation test, and its actual fertilizer effect was verified through the seed germination and cabbage heart pot test. This study has important application prospects in improving the fertilizer nutrient utilization efficiency and promoting nano-fertilizers, and also provides a scientific basis and practical basis for the design and safe utilization of new nanocarriers for controlling release and improving the nutrient absorption efficiency.

## 2. Materials and Methods

### 2.1. Materials

Glucose (Analyte Pure, Tianjin Damao, Tianjin, China), anhydrous ethanol (Analyte Pure, Tianjin Damao, China), cetyltrimethylammonium bromide (CTAB) (Analyte Pure, Tianjin Damao, China), ammonia (Analyte Pure, Tianjin Damao, China), ethyl orthosilicate (Analyte Pure, Shanghai Macklin, Shanghai, China), hydrochloric acid (Analyte Pure, Guangzhou Chemical Reagent Factory, Guangzhou, China), toluene (Analyte Pure, Guangzhou Chemical Reagent Factory, China), 3-aminopropyltriethoxysilane (Analytically Pure, Shanghai Macklin, China), dopamine hydrochloride (Analytically Pure, Shanghai Macklin, China), Tris-HCl solution (Analytically Pure, Shanghai Macklin, China), urea (99%) (Analytically Pure, Shanghai Macklin, China), trichloroacetic acid solution (10%) (Analytically Pure, Shanghai Macklin, China), and p-dimethylaminobenzaldehyde (Analytically Pure, Shanghai Macklin, China) were used.

Electric constant temperature blast drying box (DHG-9240, Shanghai Yiheng, Shanghai, China) was used to control reaction temperature, magnetic stirrer (HJ-2B, Changzhou Jintan Dadi, Changzhou, China) was used for stirring of glucose solution, electronic balance (AR224CN, Shanghai Ohaus Instrument Co., Ltd., Shanghai, China) was used for weighing of glucose, etc., low-temperature and high-speed centrifuge (5810R, Hamburg Eppendorf, Germany) was used for centrifugation of glucose, etc., vacuum drying box (DZF-6030A, Shanghai Shenxian Constant Temperature Equipment Factory, Shanghai, China) was used for drying of samples, constant temperature electric heating sleeve (CLT-1A, Shanghai LICHEN, Shanghai, China) was used for control of reaction temperature in the preparation of CN@mSiO_2_, ultrasonic cleaning machine (KQ218, Kun Shan Ultrasonic Instruments Co., Ltd., Kunshan, China) was used for ultrasonication of nanocarbon mixtures, etc., rotary mixing instrument (MX-RL-Pro, Beijing Dragon Laboratory Instruments Limited, Beijing, China) was used for mixing of CN@mSiO_2_-NH_2_ and urea, and floor constant temperature oscillator (HZQ-211C, Shanghai Yiheng, China) was used for mixing of solutions and control of temperature.

### 2.2. Preparation and Screening of Carbon Nanoparticles

**Figure 1:**
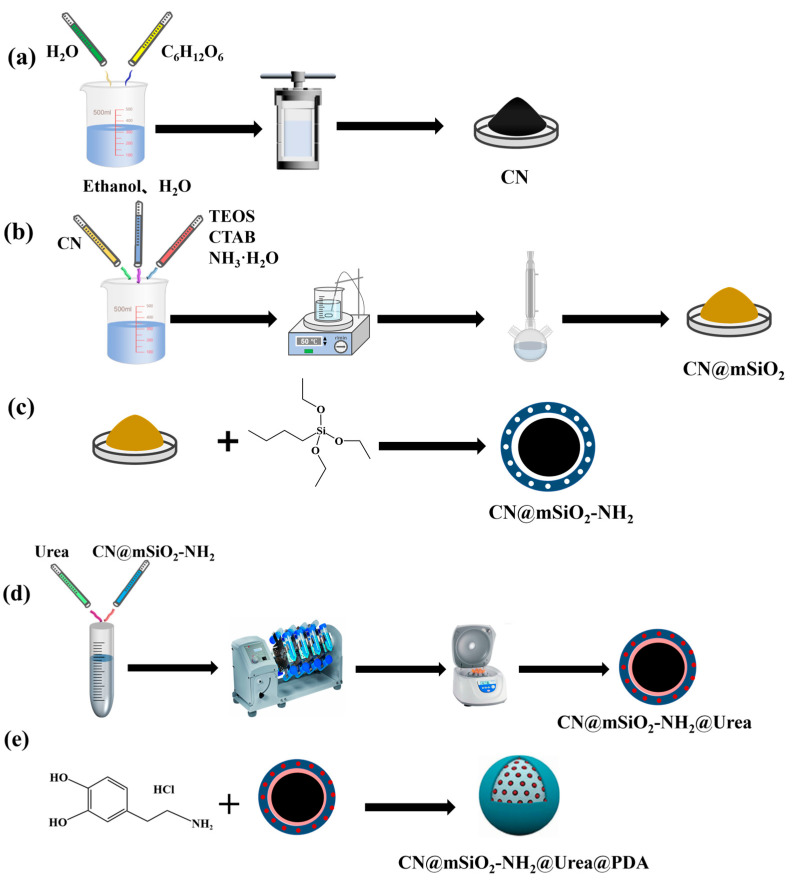
(**a**) Preparation and screening of CN; (**b**) preparation and screening of CN@mSiO_2_; (**c**) preparation and screening of CN@mSiO_2_-NH_2_; (**d**) load of urea; (**e**) preparation of CN@mSiO_2_-NH_2_@Urea@PDA.

We used the traditional hydrothermal process to generate nanocarbon (Table S1) [37]: To prepare a glucose solution with a concentration of 0.15–0.60 mol/L, dissolve a specific amount of glucose in water and stir magnetically for 20 min. Transfer the solution to a reactor, tighten the kettle cover with a wrench, and place it in an incubator at 160–190 °C for 6–12 h [38,39,40]. After the reaction is complete, cool the reactor to room temperature before opening. Centrifuged the black or brown materials and rinse three times with deionized water and ethanol, respectively, to eliminate any leftover byproducts. Produce the nanocarbon by vacuum drying at 40 °C (Figure 1a).

### 2.3. Functionalization CN@mSiO_2_ Preparation and Screening of Core–Shell-Structured Nanomaterials

Refer to the improved Stober method for CN@mSiO_2_ (Table S2) [41]. Nanocarbon was dispersed in a mixed solution with a ratio of alcohol to water of 0.4–1.0 and a concentration of ammonia of 0.11–0.21 mol/L and CTAB for 30 min by ultrasonic and magnetic stirring, respectively, and then introduced into tetraethyl orthosilicate (TEOS) (0.006–0.017 mol/L) drop by drop after continuous stirring for 10 s. The reaction mixture was stirred at 50 °C for 6 h. After the reaction was complete, the suspension was centrifuged, washed alternately three times with deionized water and anhydrous ethanol, and then dried overnight under vacuum at 60 °C to obtain CN@SiO_2_ (Figure 1b). The synthesized CN@SiO_2_ powder was dispersed in 60 mL of ethanol solution containing 6 mL of 37% HCl and refluxed at 60 °C for 3 h. The process was repeated three times, centrifuged, washed with anhydrous ethanol three times, and vacuum dried at 60 °C for 12 h to obtain CN@mSiO_2_. Then, 0.25 g of vacuum-activated CN@mSiO_2_ was weighed, 50 mL of toluene was added, and this was dispersed evenly by ultrasound. After the mixture was heated to 80 °C, 0.25 mL APTES was added drop by drop and heated at 80 °C for reflux for 4 h. After the reaction was completed, the suspension was centrifuge, the precipitation was washed with toluene three times, and then dried in vacuum at 40 °C for 12 h. The obtained product was amino-functionalized CN@mSiO_2_, i.e., CN@mSiO_2_-NH_2_ (Figure 1c).

### 2.4. Urea Load Test and Preparation of Polydopamine-Coated Nano Slow-Release Fertilizer

In order to optimize the loading rate of CN@mSiO_2_-NH_2_ on urea, a certain amount of CN@mSiO_2_-NH_2_ was added into the same urea solution of 10 mg/mL, 100 mg/mL, and 500 mg/mL, respectively. After ultrasonic dispersion for 30 min, the reaction system was vacuumed for a period of time. It was then placed in a rotary mixer at room temperature for 24 h. After the load was completed, the mixed solution was centrifuged at 12,000 rpm for 15 min, the sediment was washed with deionized water, and the product was CN@mSiO_2_-NH_2_@Urea (Figure 1d).

CN@mSiO_2_-NH_2_@Urea was uniformly dispersed in a Tris-HCl (10 mM) solution with pH = 8.5, with dopamine hydrochloride added for reaction under agitation for 20 h, centrifuged at 12,000 rpm to collect precipitation, washed with deionized water, and dried overnight in a vacuum. The resulting product was CN@mSiO_2_-NH_2_@Urea@PDA (Figure 1e).

The urea content in supernatant and washing solution was analyzed at 425 nm by UV-visible-spectrophotometer using the p-dimethylaminobenzaldehyde (DMAB) color development method [42,43]. The final loading capacity of urea was the difference between the original amount and the remaining amount. All experiments were triplicated, and the average of the results was taken as the result value.

### 2.5. Characterization

The morphology of the samples was characterized by G2 F20 S-Twin (Field Emission Transmission Electron Microscope, Peabody, MA, USA) with an accelerated voltage of 200 kV. The Fourier transform infrared spectrometer (FTIR) measurements were performed with the TENSOR 27 and measured by the KBr tablet, the powdered samples were mixed well with potassium bromide and placed in the mid-infrared region (2.5–25 um; 4000–400 cm^−1^), and the instrument resolution was adjusted to 0.5 cm^−1^ for testing. The surface elements were analyzed by K-Alpha X-ray photoelectron spectrometer. The surface charge was determined using the UK Zetasizer Nano (Malvern Panalytical, Malvern, UK) as the ZETA potential analyzer. The ULTIMA IV X-ray powder diffractometer (XRD) was used to analyze the phase and mesoporous structure of the samples, and the radiation source of the ULTIMA IV X-ray powder diffractometer is an X-ray generator with a power of 3 kW. The zero-order release kinetics equation, quasi-first-order release kinetics equation, and Higuchi diffusion equation were used to investigate the release kinetics of CN@mSiO_2_-NH_2_@Urea and CN@mSiO_2_-NH_2_@Urea@PDA nanocomposites urea. The adhesion of the composite water droplets was investigated by suspending the droplets on the silicon wafer and observing the sliding of the droplets at different tilt angles.

### 2.6. Urea Release Test

CN@mSiO_2_-NH_2_@Urea and CN@mSiO_2_-NH_2_@Urea@PDA with the same urea content were dispersed in 3 mL of demineralized water and shaken at 100 rpm/min at 25 °C. At regular intervals, 2 mL of the dispersed solution was taken and immediately replenished with fresh solution, and then the cumulative release of urea was detected by dimethylbenzaldehyde (DMAB) color development method. Finally, the zero-level release kinetic equation, quasi-level release kinetic equation, and Higuchi diffusion equation were fitted to investigate the release kinetics of urea from CN@mSiO_2_-NH_2_@Urea and CN@mSiO_2_-NH_2_@Urea@PDA.

### 2.7. Seed Germination and Pot Application of Cabbage Heart

The practical application effects of CN@mSiO_2_-NH_2_@Urea and CN@mSiO_2_-NH_2_@Urea@PDA were verified by the seed germination test and the cabbage heart pot test. The same specifications were selected as the heart seeds and the air-dry sieve was disinfected. Two layers of sterile filter paper were stacked in the washed and dried Petri dishes, 10 mL of sterile water, 0.3 g/L aqueous urea solution, and aqueous suspension of CN@mSiO_2_-NH_2_@Urea@PDA nanocomposites with the same content of urea were added, respectively, and 30 sterilized seeds of *brassica rapa* var. *chinensis* were placed in each Petri dish in a uniform and orderly manner. This was then placed in the dark culture of the incubator at 25 °C. Each treatment was repeated three times and the germination potential, germination rate, root length, germination index, and vigor index of the seeds were measured.

Germination rate = Number of germinated seeds/Number of seeds for testing × 100%Germination potential = Number of germination within the germination of test seeds/number of test seeds × 100%Germination index = ∑ Gt/D tVitality index = Mean radicle length at day 5 of germination × germination index

Here, Gt represents the number of germinated seeds on the tth day, Dt represents the corresponding germination days, and the radicle length is defined as the total length from the hypocotyl to the root tip and is directly measured using a vernier caliper with an accuracy of 0.02 mm.

Forty-nine *brassica rapa* var. *chinensis* were selected as a pot test vegetable to explore the actual nutrient supply performance of the composite material. There were no N fertilizer applications + foliar spraying water (CK), conventional N fertilizer + foliar spraying water (T1), CN@mSiO_2_-NH_2_@Urea@PDA replacement nitrogen-base fertilizer dosage 40% + foliar spraying water (T2), conventional N fertilizer application (base fertilizer added CN@mSiO_2_-NH_2_@PDA) + foliar spraying water (T3), nitrogen topdressing dosage 8.57% for foliar spraying (T4), CN@mSiO_2_-NH_2_@Urea@PDA replacement nitrogen topdressing dosage 8.57% for foliar spraying (T5), nitrogen topdressing dosage 8.57% for foliar spraying, and CN@mSiO_2_-NH_2_@PDA (T6) spraying at the same time, a total of 7 treatments. Each process was repeated 3 times. Potting tests were carried out in a thermostatic incubator at a controlled temperature of 25 °C and 80% humidity, using LED lights to simulate natural light conditions to ensure that the plants were grown in light conditions similar to the natural environment. Twenty-six days after transplanting, the agronomic traits, yield, soil nitrogen retention rate, plant nitrogen utilization rate, and nitrogen loss rate were measured.

Nitrogen utilization rate = Amount of nitrogen absorbed by the crop/Amount of nitrogen fertilizer applied × 100%Nitrogen Fertilizer Residual Rate = Amount of N left in the soil/Total amount of N fertilizer applied × 100%Nitrogen loss rate = 100 − Nitrogen utilization rate − Nitrogen fertilizer residual rate in soil layer

The experimental data were processed using Excel for basic processing and BM SPSS Statistics 29.0.1 for data processing such as significance analysis. The particle size of nanocarbon was determined using NanoMeasurer +1.2.5 to determine the particle size of nanocarbon. Graphing and equation fitting were performed using Origin 2021.

## 3. Results

### 3.1. Preparation and Screening of Nanocarbon

#### Effect of Glucose Concentration, Reaction Time, and Temperature on Nanocarbon and Its Structural Characterization

**Figure 2:**
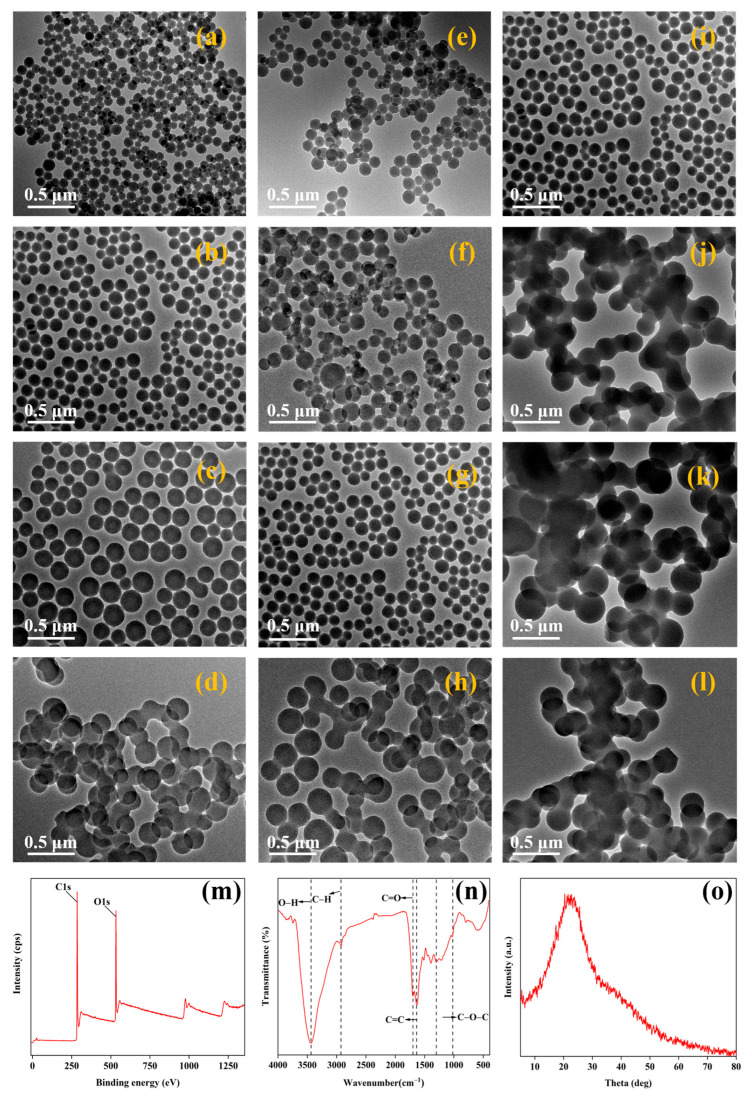
The TEM images of the nanocarbon glucose concentrations of (**a**) 0.15, (**b**) 0.3, (**c**) 0.45, and (**d**) 0.6 mol/L, reaction times of (**e**) 6, (**f**) 8, (**g**) 10, and (**h**) 12 h, reaction temperatures of (**i**) 160, (**j**) 170, (**k**) 180, and (**l**) 190 °C; (**m**) XPS, (**n**) FTIR, and (**o**) XRD spectra of nanocarbon fabricated under optimal process conditions.

Transmission electron microscopy TEM was used to explore the effects of the glucose concentration, reaction time, and temperature on nanocarbon. When the reaction temperature was kept at 160 °C, the reaction time was 10 h, and the glucose concentration was 0.15, 0.30, 0.45, and 0.60 mol/L, respectively (Figure 2a–d), and the average particle sizes of nanocarbon were 71, 96, 170, and 162 nm, respectively (Table S3). It showed that the particle size of nanocarbon increased with the increase in the glucose concentration at a low concentration; when the concentration was increased from 0.45 mol/L to 0.60 mol/L, the particle size of nanocarbon became smaller. With the increase in the glucose concentration, the cross-linking reaction caused by intermolecular dehydration between the oligosaccharides and the formation of nuclei in the solution of isotropic growth leads to an increase in the viscosity of the solution, limiting its movement in the solution, which leads to the decrease in the size of the nanocarbon particles [44] which could also be attributed to the fact that the high concentration of glucose increased the collision of the nanocarbon formed, and the nanocarbon particles began to show serious agglomeration [45]. Considering the optimal dispersion properties of the nanocarbon and the small size required, the optimal concentration of the glucose solution was determined to be 0.30 mol/L in this work.

When the reaction temperature was kept at 160 °C and the concentration of glucose was 0.30 mol/L, the average particle size of nanocarbon increased from 75 nm to 157 nm (Table S4) as the reaction time increased from 6 h to 12 h (Figure 2e–h). As the reaction time increases, glucose continues to decompose and intermediate products are preferentially adsorbed on the surface of existing nanocarbon, which explains the nucleation and growth process of nanocarbon. However, too short a reaction time leads to the generation of small-sized nanocarbon, whose relatively high surface activity results in the agglomeration of the nanocarbon [46]; too long a reaction time results in continuous changes in the formed carbon spheres, and the cross-linking phenomenon between the spheres leads to agglomeration. Therefore, nanocarbon with reasonable dispersibility can be obtained when the reaction time is appropriate. Considering the dispersibility of nanocarbons and their average particle size, the optimum reaction time was determined to be 10 h in this work.

The transmission electron microscopy images and average particle size of samples carbonated with a glucose solution at a concentration of 0.30 mol/L for 10 h when the reaction temperature ranged from 160 to 190 °C showed that the morphology and particle size of the prepared nanocarbon were significantly affected by the hydrothermal temperature. When hold reaction time is 10 h, the glucose concentration is 0.30 mol/L; as the reaction temperature increased from 160 °C to 190 °C, the average particle size of the nanocarbon increased from an initial 96 nm to 332 nm, but then decreased to 245 nm (Table S5). In general, the higher the reaction temperature, the larger the particle size of the product [47]. However, the formation of carbonaceous spheres was generally dominated by nucleation and growth mechanisms, and at higher reaction temperatures (Figure 2l), the driving force of the hydrothermal process and the rate of carbon nucleation were high, which led to the simultaneous occurrence of nucleation and growth of carbonaceous spheres, resulting in the formation of smaller carbon nanoparticles. In addition, the spherical nanocarbon obtained at lower temperatures had less agglomeration and the least agglomeration (Figure 2i). Therefore, the optimum reaction temperature in this work was 160 °C.

XPS, FTIR, and XRD were used to explore the structural changes and elemental distribution of nanocarbon (Figure 2m–o). The results show that there were only two elements, C and O, in the nanocarbon, and the content of the C element was higher than that of the O element, indicating that the nanocarbon was dominated by the C element and contained a large number of oxygen-containing groups (Figure 2m). A large number of oxygen-containing functional groups were exposed on the surface of the nanocarbon. The broadening peak at 3436 cm^−1^ was attributed to the O-H stretching vibration in hydroxyl or carboxyl groups, indicating the presence of -OH groups on the surface of the nanocarbon. The peak at 2925 cm^−1^ corresponds to the peak of the stretching vibration of the C-H in aliphatic or alkyl groups in the carbonyl group. The peak at 1636 cm^−1^ corresponds to the stretching vibration of the olefinic C=C, verifying the presence of the carbonyl group. The peak of C=O stretching vibration in the aromatic ring (carbonyl, ester group, and carboxyl group) was at 1699 cm^−1^ [48]. Absorption peaks at 1000–1500 cm^−1^ correspond to antisymmetric stretching vibration and symmetric stretching vibration of aromatic C-O-C. The presence of aromatic rings on the surface of the nanocarbon also provides supportive evidence for the hydrolysis and aromatization of glucose during hydrothermal processes, and the presence of functional groups -OH, C=O, and -COOH improves the hydrophilicity of the nanocarbon as well as its dispersion and stability in aqueous solution, which was the basis for its further surface functionalization applications (Figure 2n). The XRD spectrum shows that the diffraction peaks of nanocarbon appeared around 20–25° (2θ) with a broad peak shape, in addition to the absence of other characteristic peaks, indicating that nanocarbon contains a large amount of disordered structural carbon and amorphous carbon existed in the nanocarbon [49,50] (Figure 2o).

### 3.2. Preparation and Characterization of Functionalization CN@mSiO_2_ Core–Shell-Structured Nanomaterials

#### 3.2.1. Effect of TEOS Concentration, Ammonia Concentration, and Alcohol–Water Ratio on CN@mSiO_2_ Core–Shell-Structured Nanomaterials

**Figure 3:**
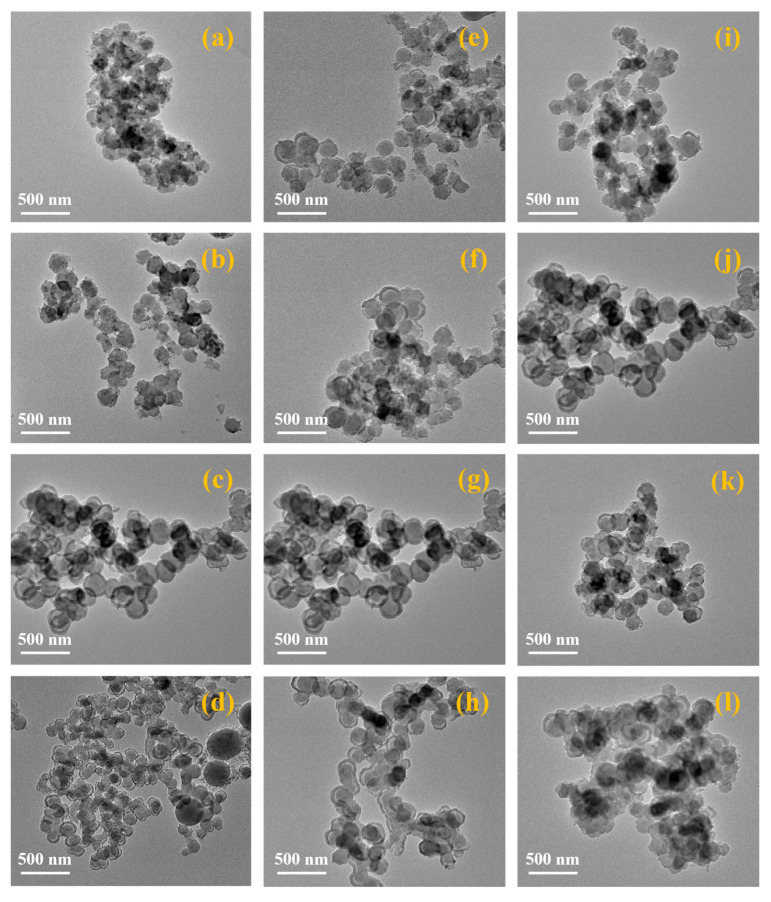
The TEM images of the surface of the CN@mSiO_2_ composite nanoparticles with TEOS concentrations of (**a**) 0.006, (**b**) 0.011, (**c**) 0.017, and (**d**) 0.023 mol/L; ammonia concentrations of (**e**) 0.11, (**f**) 0.15, (**g**) 0.18, and (**h**) 0.21 mol/L; and alcohol–water ratios of (**i**) 0.4, (**j**) 0.6, (**k**) 0.8, and (**l**) 1.0.

The surface morphology of CN@mSiO_2_ composite nanoparticles at different TEOS concentrations, ammonia concentrations, and alcohol–water ratios were observed by the TEM method (Figure 3a–l). When the alcohol–water ratio was kept at 0.6, the ammonia concentration was 0.18 mol/L, and the TEOS concentration was 0.006–0.011 mol/L (Figure 3a,b), it was difficult to generate a complete mSiO_2_ shell layer on the surface of nanocarbon, and CN@mSiO_2_ composite nanoparticles with a core–shell structure could not be synthesized. For the TEOS concentration of 0.017–0.023 mol/L (Figure 3c,d), the mSiO_2_ shell layer was successfully encapsulated on the surface of nanocarbon, the thickness of the shell layer gradually increased and became more uniform with the increase in the TEOS concentration, and the surface was smoother. However, when the TEOS concentration was too high, it was difficult for it to fully aggregate and grow on the surface of the nanocarbon particles due to the accelerated hydrolysis rate, leading to the generation of excessive mSiO_2_ primary particles, which resulted in the generation of more mSiO_2_ nanoparticles without cores. Meanwhile, the specific surface area and total pore volume of the CN@mSiO_2_ composite nanoparticles increased with the increase in the TEOS concentration (Table S6), and the pore diameter of the composite particles increased with the increase in the TEOS concentration when the TEOS concentration was in the range of 0.006–0.017 mol/L, and the pore diameter of the composite particles increased with the increase in the TEOS concentration when the TEOS concentration was in the range of 0.017–0.023 mol/L; the pore size of the composite particles was basically unchanged. This was due to the fact that during the hydrolysis and condensation of TEOS, the thickness of the mSiO_2_ shell layer gradually increased with the increase in the TEOS concentration, and the secondary mesopores inside the nano-shell layer were accompanied by an increase in the size due to the presence of ordered liquid crystal templates from CTAB micelles until a certain concentration limit. Based on the results shown in Figure 2 and Table S6, 0.017 mol/L was adopted as the optimum concentration of TEOS in this study.

When the alcohol–water ratio was kept at 0.6, the TEOS concentration was 0.017 mol/L (Table S7), and the ammonia concentration was 0.11 and 0.15 mol/L (Figure 3e,f). The CN@mSiO_2_ composite nanoparticles appeared to be incompletely encapsulated and when the ammonia concentration was 0.18 and 0.21 mol/L (Figure 3g,h), the prepared CN@ mSiO_2_ composite particles were basically core–shell structures, but there was some agglomeration in the nanocarbon cores. This may be due to the ammonia concentration affecting the surface potential of colloidal particles and the ionic strength of the reaction solution. When the ammonia concentration was too low, the ionic strength of the reaction solution was low, the surface potential of the mSiO_2_ primary particles was high, and the electrostatic repulsion was strong, which reduces the chance of collision with nanocarbon and makes it difficult for nanocarbon to capture the mSiO_2_ primary particles. When the ammonia concentration increased to 0.21 mol/L (Figure 3h), the ionic strength in the reaction solution was too high, and the nanocarbon was destabilized and agglomerated before being encapsulated by the mSiO_2_, resulting in the phenomenon of multiple nanocarbon particles encapsulated by the mSiO_2_.

When the ammonia concentration was in the range of 0.11–0.21 mol/L (Figure 3e–h), the specific surface area of the CN@mSiO_2_ composite nanoparticles increased with the increase in the ammonia concentration, while the total pore volume and pore size did not change much. The increase in the ammonia concentration will accelerate the hydrolysis reaction rate of TEOS, and more generated mSiO_2_ particles will gather on the surface of nanocarbon, resulting in the thickening of the shell layer, the increase in the particle size, and the increase in the specific surface area. Based on the morphology of CN@mSiO_2_ composite nanoparticles and the specific surface area, pore volume, and pore size, 0.18 mol/L was the optimal concentration of ammonia, and the specific surface area was 481.1 m^2^/g, pore volume was 0.51 m^3^/g, and pore size was 2.36 nm.

When the ammonia concentration and TEOS concentration were maintained at 0.18 mol/L and 0.017 mol/L (Table S8), respectively, and the alcohol–water ratio was 0.4, a certain amount of mSiO_2_ particles without cores appeared, and an agglomeration phenomenon occurred (Figure 3i); when the alcohol–water ratio was 0.6, the composite particles were regular in shape, tended to be spherical, and the dispersibility was better (Figure 3j); when the alcohol–water ratio was 0.8, the surface irregular composite particles began to increase, and the agglomeration phenomenon was aggravated (Figure 3k); and when the alcohol–water ratio was further increased to 1.0, a large number of surface irregular composite particles and the agglomeration phenomenon was serious (Figure 3l). The polarity of the reaction solution was closely related to its water content, and a high alcohol–water ratio will lead to a decrease in the polarity of the reaction solution, which in turn reduces the stability of the carbon nanoparticles, and a certain degree of agglomeration occurs; when the alcohol–water ratio was too low, the hydrolysis of TEOS was too fast, resulting in a large number of small-sized mSiO_2_ primary particles, which were difficult to completely attach to the surface of the carbon nanoparticles, and some of which grew to be the stable mSiO_2_ secondary particles on their own. A suitable alcohol–water ratio will make the stability of carbon nanoparticles in the reaction solution increase, tend to generate regular-shaped, smooth-surface spherical composite particles, and reduce the agglomeration.

Since CTAB can spontaneously form structurally ordered micelles in water, it was often used as a mesoporous template in the synthesis of mesoporous silica, and its state in solution intuitively affects the whole reaction process. Among them, the appropriate amount of EtOH organic solvent with a certain polarity can regulate the formation process of CTAB micelles and then regulate its mesoporous structure, so the alcohol–water ratio was an important factor affecting the mesoporous structure of CN@mSiO_2_. There was no obvious pattern for the effect of the alcohol–water ratio on the mesoporous structure and specific surface area of CN@mSiO_2_, but when the alcohol–water ratio was 0.6, the results of the specific surface area, total pore volume, and pore size of CN@mSiO_2_ composite nanoparticles were optimal, which were 481.1 m^2^/g, 0.51 cm^3^/g, and 2.36 nm, respectively, suggesting that the CTAB was able to form optimal micellar structures through self-assembly at this alcohol–water ratio. The micelle-like structure at this alcohol–water ratio is shown in Table S6. In summary, the optimal alcohol–water ratio for preparing CN@mSiO_2_ composite nanoparticles was 0.6.

#### 3.2.2. CN@mSiO_2_-NH_2_ Preparation and Characterization of Core–Shell-Structured Nanomaterials

**Figure 4:**
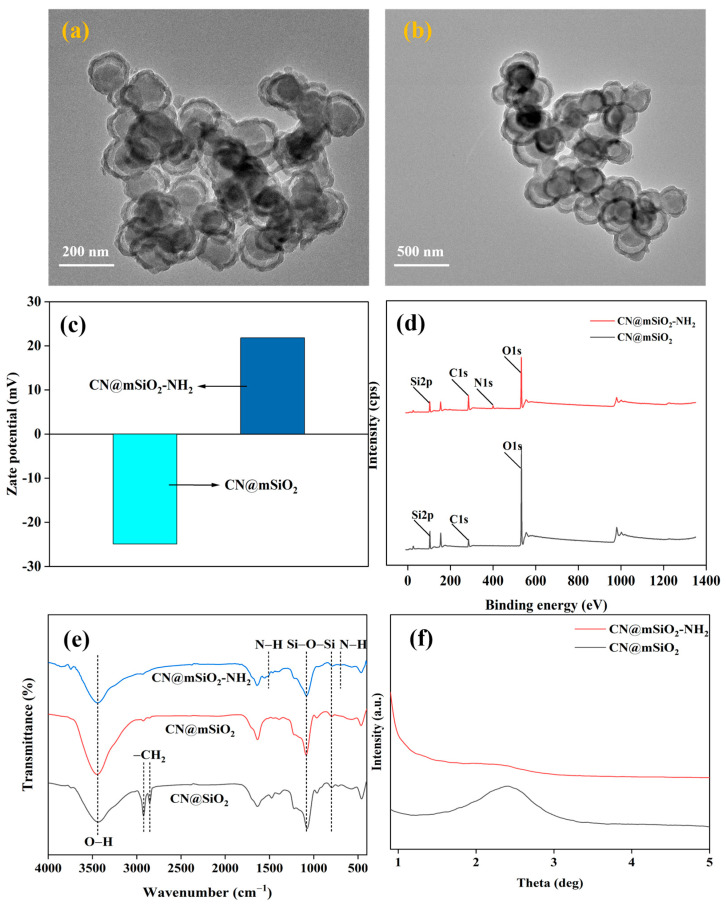
TEM images of (**a**) CN@mSiO_2_ and (**b**) CN@mSiO_2_-NH_2_, (**c**) zeta potentials, (**d**) XPS spectra, (**e**) FTIR spectra, and (**f**) XRD spectra of CN@mSiO_2_ and CN@mSiO_2_-NH_2_.

The amino-modification of CN@mSiO_2_ prepared under optimal process conditions was carried out in a toluene medium using APTES, and CN@mSiO_2_-NH_2_ was obtained by centrifugal separation and vacuum drying. The surface morphology of CN@mSiO_2_ and CN@mSiO_2_-NH_2_ was analyzed using TEM photographs (Figure 4a,b). The CN@mSiO_2_ composite nanoparticles prepared under the optimal parameters showed a core–shell structure, indicating that the nanocarbon was successfully encapsulated by the mSiO_2_ shells, and the outer layer presented a rough structure which tended to be spherical and porous with a particle size of about 200 nm; the CN@mSiO_2_-NH_2_ composite nanoparticles, on the other hand, presented a more regular, smoother surface with improved dispersion (Figure 4b).

The Zeta potential was used to characterize the surface potential of the composite nanoparticles (Figure 4c). The ζ-potential of CN@mSiO_2_ was measured at −24.9 mV, attributed to the presence of abundant silanol groups on its surface. Following amino modification, the CN@mSiO_2_-NH_2_ sample showed an increased ζ-potential of 21.80 mV, likely due to the interaction between the grafted amino groups and H^+^ ions from water dissociation, rendering the surface positively charged. Additionally, the XPS was used to determine the elemental composition of the CN@mSiO_2_ and CN@mSiO_2_-NH_2_ (Figure 4d). CN@mSiO_2_ exhibited characteristic peaks for C, O, and Si at 295.08 eV, 532.84 eV, and 103.62 eV, respectively. In contrast, CN@mSiO_2_-NH_2_ displayed a weak N peak at 399.61 eV, confirming the successful grafting of amino groups onto the CN@mSiO_2_ surface.

The structure of each product composite was analyzed by FTIR (Figure 4e). The Si-O-Si antisymmetric stretching vibrational peaks at a 1083 cm^−1^ wavelength, the Si-O-Si symmetric stretching vibrational peaks at an 800 cm^−1^ wavelength, the Si-O-Si bending vibrational peaks at a 460 cm^−1^ wavelength, and O-H stretching vibrational peaks at a 3440 cm^−1^ wavelength were recorded. The successful capping of SiO_2_ was confirmed, which was in agreement with the TEM characteristics results. The strong aliphatic peaks at 2922 cm^−1^ and 2852 cm^−1^ were attributed to the stretching vibration of the single-bond CH_2_, suggesting that CTAB was present in CN@SiO_2_. However, the above peaks were almost absent in CN@mSiO_2_, indicating that CTAB was largely removed from CN@mSiO_2_. There was a very weak peak at 698 cm^−1^ and 1510 cm^−1^ each, corresponding to the bending vibration of the N-H bond and the -NH^3+^ symmetry vibration peak, indicating that the amino group has been successfully modified onto CN@mSiO_2_, which was in agreement with the XPS data.

The structure and orderliness of the mesopores in the nanocomposite were characterized by XRD analysis (Figure 4f). Prior to modification, CN@mSiO_2_ exhibited a weak and broad peak at approximately 2.5°, corresponding to the (100) crystal plane, which was a hallmark feature of hexagonal mesoporous materials. The relatively low intensity of this peak suggests that while the sample possesses a mesoporous structure, its degree of order was limited. Upon the introduction of amino groups, the diffraction peak associated with the (100) plane diminished significantly. This reduction can be attributed to the amino groups occupying the pore channels, thereby decreasing the scattering contrast between the pore walls and the pore channels.

### 3.3. CN@mSiO_2_-NH_2_ Applications in Fertilizers

#### 3.3.1. Loading Properties of Urea, Preparation, and Structure of CN@mSiO_2_-NH_2_@Urea@PDA Nano Slow-Release Fertilizer

**Figure 5:**
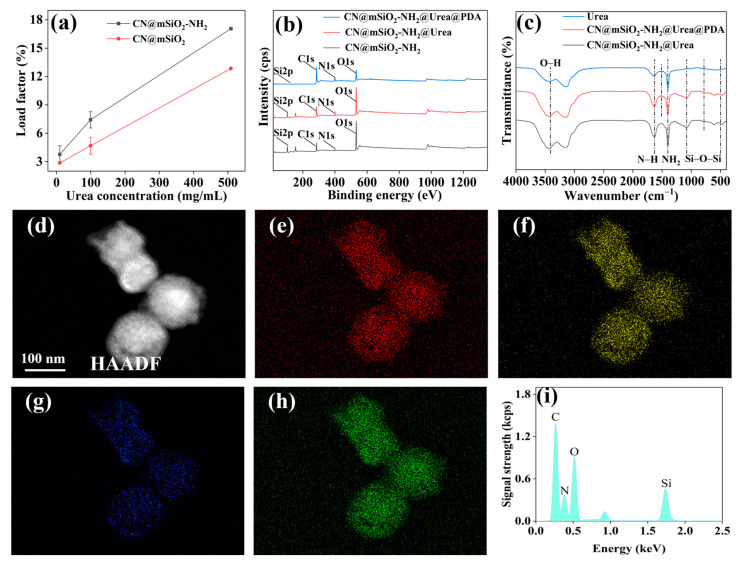
(**a**) Effect of urea concentration on urea loading rate of different composite nanoparticles; (**b**) XPS spectra of different composite nanoparticles and (**c**) FTIR spectra; (**d**) The high-angle annular dark-field STEM of CN@mSiO_2_-NH_2_@Urea@PDA; (**e**) The carbon distribution in CN@mSiO_2_-NH_2_@Urea@PDA in the EDS-mapping; (**f**) The oxygen distribution in CN@mSiO_2_-NH_2_@Urea@PDA in the EDS-mapping; (**g**) The silicon distribution in CN@mSiO_2_-NH_2_@Urea@PDA in the EDS-mapping; (**h**) The nitrogen distribution in CN@mSiO_2_-NH_2_@Urea@PDA in the EDS-mapping; (**i**) EDS spectra of analysis for the CN@mSiO_2_-NH_2_@Urea@PDA.

The loading rates of CN@mSiO_2_ (Figure 5a) and CN@mSiO_2_-NH_2_ (Figure 5b) increase with the concentration of the urea solution. At a urea concentration of 10 mg/mL, the maximum loading rates for CN@mSiO_2_ and CN@mSiO_2_-NH_2_ were only 2.87% and 3.77%, respectively. However, when the concentration of the urea solution increases to 500 mg/mL, the maximum loading rates for CN@mSiO_2_ and CN@mSiO_2_-NH_2_ rise to 12.85% and 17.06%, respectively. Therefore, in this study, a urea concentration of 500 mg/mL was determined as the optimal loading concentration for nanoparticles. Additionally, when replacing CN@mSiO_2_ with CN@mSiO_2_-NH_2_ at the optimal urea concentration, the loading rate of CN@mSiO_2_-NH_2_ significantly increased. This confirms that the introduction of amino functional groups can significantly enhance the loading performance of CN@mSiO_2_ for urea. This improvement was attributed to the fact that CN@mSiO_2_-NH_2_ possesses amino functional groups similar to those of urea, potentially leading to the crystallization of urea. During the crystallization process of urea, the oxygen center of urea forms hydrogen bonds with two additional N-H groups from two independent urea molecules, forming a network of urea crystals. The amino groups on CN@mSiO_2_-NH_2_ may serve as seeds for the growth of urea crystals. Compared to the silanol seed sites on CN@mSiO_2_, the amino group seed sites on CN@mSiO_2_-NH_2_ were easier to access, resulting in higher seeding efficiency and increased urea loading.

CN@mSiO_2_-NH_2_@Urea nanocomplexes were prepared by loading urea into CN@mSiO_2_-NH_2_ under vacuum conditions. Subsequently, polydopamine was encapsulated in the outer layer of CN@mSiO_2_-NH_2_@Urea using the oxidative self-polymerization of dopamine hydrochloride under alkaline conditions to finally obtain the CN@mSiO_2_-NH_2_@Urea@PDA nano-slow-release fertilizer.

The surface elemental compositions of CN@mSiO_2_-NH_2_, CN@mSiO_2_-NH_2_@Urea, and CN@mSiO_2_-NH_2_@Urea@PDA were analyzed using XPS (Figure 5b). Compared to CN@mSiO_2_-NH_2_, the XPS energy spectra of the remaining two showed a significantly enhanced characteristic peak of elemental N at 399.61 eV, which was attributed to the successful loading of urea. The slight increase in the intensity of O1s in CN@mSiO₂-NH₂@Urea compared to CN@mSiO₂-NH₂@Urea was attributed to the enhancement of the oxygen content on the surface as a result of the introduction of urea and the increase in the intensity of the peaks of N1s and C1s in CN@mSiO₂-NH₂@Urea@PDA compared to the previous two samples. This indicates that the introduction of the PDA coating significantly changed the chemical composition of the sample surface, especially the content of the carbon and nitrogen elements. Meanwhile, the C element peaks are stronger than the O element peaks, which is due to the large amount of carbon in polydopamine, confirming the successful coating of the polydopamine layer.

The bond interactions of urea in the matrix structure were analyzed using FTIR (Figure 5c). In the FTIR spectra of CN@mSiO_2_-NH_2_@Urea and CN@mSiO_2_-NH_2_@Urea@PDA, the absorption peaks at 1080 and 470 cm^−1^ were attributed to the asymmetric stretching vibration of Si-O-Si, and the peak at 783 cm^−1^ was attributed to the symmetric stretching of Si-O-Si, in addition to which 3413 cm^−1^ could be attributed to the Si-OH stretching vibration, which confirms the presence of the SiO_2_ framework. By comparing the IR spectra of urea, the presence of urea can be observed at 1629 cm^−1^, which corresponds to the δ-deformation of NH_2_, whereas a bending vibrational peak corresponding to N-H can be observed at 1401 cm^−1^, and the strong bending vibration of N-H suggests that the amine group from urea may be bound to the amine point of the aminopropyl group on the surface of CN@mSiO_2_-NH_2_, which corresponds to the amine group of CN@ mSiO_2_-NH_2_ higher-urea loading. The characteristic peak of the indole structure at 1510 cm^−1^ appeared after coating with PDA.

TEM was used to observe the surface morphology of CN@mSiO_2_-NH_2_, CN@mSiO_2_-NH_2_@Urea, and CN@mSiO_2_-NH_2_@Urea@PDA (Figure S1a–c). As can be seen from the figure, the contrast between the CN@mSiO_2_-NH_2_@Urea core–shell interstitial voids and the outer shell was significantly reduced compared to CN@mSiO_2_-NH_2_, indicating that the urea was successfully loaded. The shell layer of CN@mSiO_2_-NH_2_@Urea@PDA was significantly thicker, the outer layer became rougher due to the shell being encapsulated by polydopamine, the pore space was blocked by PDA, and porosity was reduced, which was conducive to slowing down the release of urea.

The elemental species and contents of CN@mSiO_2_-NH_2_@Urea (Figure S1d–i) and CN@mSiO_2_-NH_2_@Urea@PDA (Figure 5d–i) were analyzed using graphical EDS spectroscopy. EDS spectra indicate that both contain the elements of C, N, O, and Si. The Si was mainly derived from the mSiO_2_ shell layer, which shows a distinctive profile (Figure 5g); the appearance of the characteristic peaks of the element N confirms that the urea has been successfully loaded (Figure 5h,i). Compared with CN@mSiO_2_-NH_2_@Urea, the carbon distribution in the outer layer of CN@mSiO_2_-NH_2_@Urea@PDA was significantly higher (Figure 5e and Figure S1e), and the proportion of the C element was significantly higher than that of the O element (Figure 5i and Figure S1i), which was due to the additional C and O elements provided by the PDA layer. C was the dominant element which further confirms that PDA has been successfully encapsulated, and also coincides with the XPS data.

**Figure 6:**
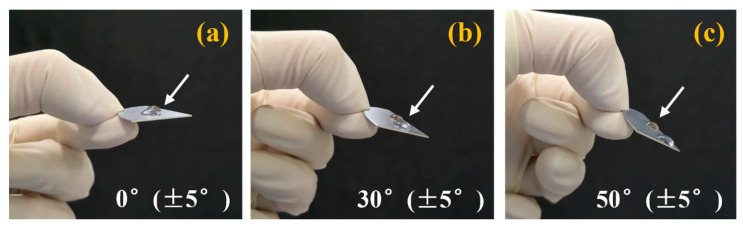
Images of urea and CN@mSiO_2_-NH_2_@Urea@PDA droplet roll-off on the silicon chip surface at (**a**) 0°, (**b**) 30°, and (**c**) 50°tilt angles.

The adhesion properties of CN@mSiO_2_-NH_2_@Urea@PDA were explored using the slippage of urea and CN@mSiO_2_-NH_2_@Urea@PDA droplets on hydrophobic silicon wafers at different angles (Figure 6a–c). Both were in equilibrium when the tilt angle of the hydrophobic silicon wafer was zero; when the tilt angle of the hydrophobic silicon wafer was 30° (±5°), the urea droplets started to undergo slippage. In contrast, even when the tilt angle of the hydrophobic silicon wafer reached 50° (±5°), the CN@mSiO_2_-NH_2_@Urea@PDA droplets did not undergo slippage. This demonstrates that the adhesion of CN@mSiO_2_-NH_2_@Urea@PDA was significantly improved. But the adhesion of PDAs plays out differently depending on the humidity level and temperature level, and some studies have shown that the adhesion of PDA remains strong in water [51].

SEM was used to explore the adhesion properties of CN@mSiO_2_-NH_2_@Urea (Figure S2a–d) and CN@mSiO_2_-NH_2_@Urea@PDA (Figure S2e–h) droplets after drying. Both CN@mSiO_2_-NH_2_@Urea and CN@mSiO_2_-NH_2_@Urea@PDA particles without distilled water rinsing were densely distributed, and after a large amount of distilled water rinsing several times, only small particles of CN@mSiO_2_-NH_2_@Urea were left to be sparsely distributed on the hydrophobic wafers, in contrast to CN@mSiO_2_-NH_2_@Urea@PDA particles remaining on the hydrophobic silicon wafer in large quantities, and its adhesion performance was significantly enhanced. This was due to the good adhesion property of PDA, and the large number of catechol groups present in PDA can interact with the silicon hydroxyl groups on the surface of the silicon wafer through hydrogen bonding, thus preventing the particles from slipping off.

#### 3.3.2. Release Periods of CN@mSiO_2_-NH_2_@Urea and CN@mSiO_2_-NH_2_@Urea@PDA

**Figure 7:**
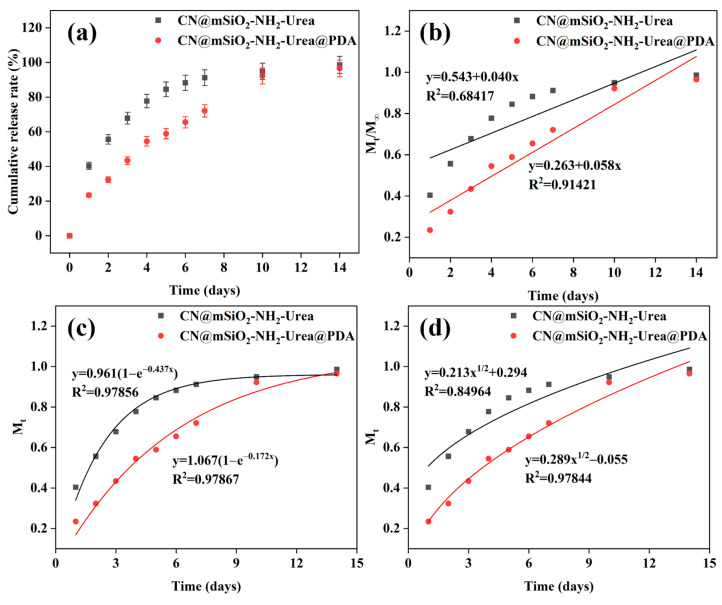
(**a**) Urea release profiles from CN@mSiO_2_-NH_2_@Urea and CN@mSiO_2_-NH_2_@Urea@PDA; Urea release profiles of (**b**) zero-level release model, (**c**) quasi-level release model, and (**d**) Higuchi model.

To investigate the release period of urea from CN@mSiO_2_-NH_2_@Urea and CN@mSiO_2_-NH_2_@Urea@PDA, it was verified by placing them in water to test the dissolution rate (Figure 7a). After 14 days of retardation, the release rate of CN@mSiO_2_-NH_2_@Urea with unblocked mesopores was relatively fast, and about 78% of the urea was released within 4 days, whereas the grafted PDA with its mesopores blocked restricted the release of urea, and the cumulative release rate of urea decreased to 54% within 4 days, with a significant improvement in the retardation performance. This was due to the fact that the release efficiency of urea depends on the rate at which water molecules penetrate into the carrier pores and lumen and dissolve the urea adsorbed on the channels and in the lumen and then diffuse. On the one hand, the presence of a large number of solid-phase interactions between the urea molecules and the carrier reduces the diffusion of the urea; on the other hand, the blocking of the polydopamine layer makes the force that needs to be overcome for the diffusion of the dissolved urea stronger, and, as a result, the CN@mSiO_2_-NH_2_@Urea@PDA has a better slow-release effect.

Three mathematical models (the zero-level release model, quasi-level release model, and Higuchi model) were used to explore the release mechanism of urea (Figure 7b–d). For urea release from CN@mSiO_2_-NH_2_@Urea and CN@mSiO_2_-NH_2_@Urea@PDA, the best fit of the data was obtained using the quasi-primary release model fit, and the release of urea was more in line with the quasi-primary release model, with the correlation coefficients of the release rate of urea being 0.97856 and 0.97867, respectively (Figure 7c). Therefore, the urea release process in CN@mSiO_2_-NH_2_@Urea and CN@mSiO_2_-NH_2_@Urea@PDA was mainly a diffusion mechanism controlled by the concentration gradient.

### 3.4. Evaluation of CN@mSiO_2_-NH_2_@Urea@PDA for Practical Applications

#### 3.4.1. Seed Germination Assay

**Figure 8:**
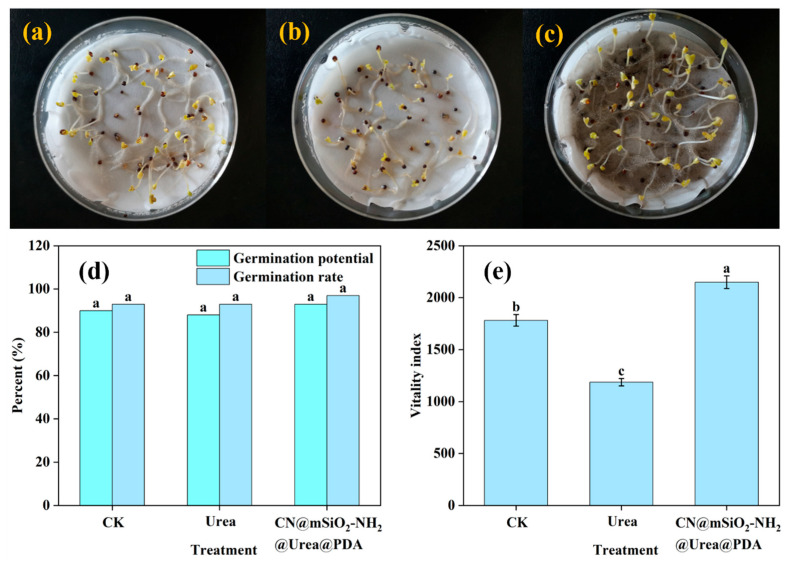
Imagery of different treatment seed germination processes: (**a**) CK, (**b**) Urea, (**c**) and CN@mSiO_2_-NH_2_ @Urea@PDA; (**d**) germination potential and germination rate, and (**e**) vitality index of *Brassica rapa* var. *chinensis* after 5 days of incubation in different treatments. Note: Different letters represent significant differences between treatments (*p* < 0.05).

A seed germination test was used to investigate the quality and vitality of seeds after the application of CN@mSiO_2_-NH_2_@Urea@PDA (Figure 8a–e). The addition of urea and CN@mSiO_2_-NH_2_@Urea@PDA did not produce any significant effect (*p* < 0.05) on the germination potential and germination percentage of cauliflower seeds (Figure 8d). Notably, the viability index of cauliflower seeds was significantly higher than that of the control group (Figure 8e) after the addition of CN@mSiO_2_-NH_2_@Urea@PDA (Table S9). On the one hand, this may be due to the fact that the carrier slowed down the release of urea, and the concentration of urea in the Petri dish did not reach the concentration that inhibited the growth of the seeds; on the other hand, CN@mSiO_2_-NH_2_@Urea@PDA increased the ability of water capture and retention during seed germination, and enhanced the antioxidant enzyme activity and root vitality of the seeds. This therefore promotes the growth of Cauliflower seeds, and CN@mSiO_2_-NH_2_@Urea@PDA has good biosafety.

#### 3.4.2. Pot Experiment

**Figure 9:**
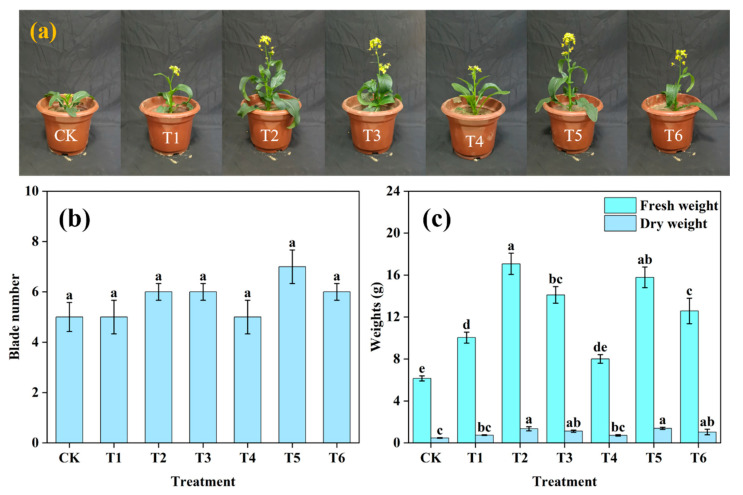
(**a**) Graph of *brassica rapa* var. *chinensis* cultivated under different treatments; (**b**) blade number and (**c**) dry and fresh weight.

The practical application of CN@mSiO_2_-NH_2_@Urea@PDA was explored by using a pot experiment of *Brassica rapa* var. *chinensis* (Figure 9a–c). *Brassica rapa* var. *chinensis* cultivated with CN@mSiO_2_-NH_2_@Urea@PDA showed better growth (Tables S10 and S11). The application of nitrogen fertilizer affected the growth effect of the *brassica rapa* var. *chinensis*, and the T5 treatment had a more significant effect on the number of leaves of *brassica rapa* var. *chinensis* compared with the blank treatment (Figure 9b). Compared with the other treatments, the fresh weight of *brassica rapa* var. *chinensis* in the T2 treatment was significantly better than the control group, which increased by 69.94% compared with the conventional fertilizer application, followed by the T5 treatment, which increased by 57.10% (Figure 9c). The effect of the CN@mSiO_2_-NH_2_@Urea@PDA on the growth of *brassica rapa* var. *chinensis* plants was, on the one hand, due to its nitrogen slow-release effect, which could provide a stable supply of nitrogen during the nitrogen-demanding period of *brassica rapa* var. *chinensis* and promote the organ differentiation, formation, and increase in the plant biomass; on the other hand, it was due to the fact that its nanocarbon could affect the expression of certain functional protein genes in the body of the plant and enhance the photosynthetic activity of the chloroplasts in the leaves of the plant, which could promote the leaf blade growth. The above experimental results proved that compared with ordinary urea, both the soil and foliar application of CN@mSiO_2_-NH_2_@Urea@PDA can effectively promote the growth and increase the yield of *brassica rapa* var. *chinensis*.

Different nitrogen fertilizer treatments yield varying nitrogen utilization efficiencies in choy sum (Table S12). Compared to T1, the nitrogen utilization efficiency of choi sum significantly increased after T2 and T5 treatments (*p* < 0.05), with the T2 treatment achieving the highest nitrogen utilization efficiency of 21.64%, an increase of 15.07% compared to the T1 treatment. And the nitrogen utilization rate of the T5 treatment was 18.37%. Sun et al. used 90 L ha^−1^ wood vinegar as a foliar fertilizer, with the nitrogen utilization havin only a 14.92% increase [52]. The statistical results indicated that the T4, T5, and T6 treatments, involving foliar nitrogen fertilizer spraying, resulted in varying degrees of decrease in the soil nitrogen residue rates compared to T1. Compared to T1, the soil nitrogen residue rates significantly increased in the T2 and T3 treatments, with the T2 treatment having the highest soil nitrogen residue rate at 56.15%. Among all treatments, T2 had the lowest nitrogen loss rate at 22.21%, a reduction of 25.42% compared to the T1 treatment; among foliar treatments, T5 had the lowest nitrogen loss rate at 37.09%, a decrease of 10.54% compared to the T1 treatment. The above results suggest that, compared to a conventional urea application, the combined application of CN@mSiO_2_-NH_2_@Urea@PDA in soil was beneficial for choy sum’s nitrogen absorption and utilization, aided in retaining residual nitrogen in the soil, and reduced nitrogen loss; the foliar spraying of CN@mSiO_2_-NH_2_@Urea@PDA was beneficial for enhancing choy sum’s nitrogen utilization efficiency and minimizing nitrogen volatilization losses. Compared to the previous single slow-release fertilizers and foliar fertilizers, CN@mSiO_2_-NH_2_@Urea@PDA can be used both as a slow-release fertilizer for application in soil and as a foliar fertilizer for spraying on leaves. It not only reduces fertilizer usage, but also alleviates environmental problems caused by low utilization rates, demonstrating a certain practical application value.

## 4. Conclusions

In this study, core–shell nanocarbon-based slow-release foliar fertilizer (CN@mSiO_2_-NH_2_@Urea@PDA) was prepared with the core of nanocarbon prepared by hydrothermal method. The BET (84.1 m^2^/g and 0.26 cm^3^/g changed to 23.8 m^2^/g and 0.14 cm^3^/g) indicated that the pore space and the surface of the material had been encapsulated successfully by PDA, confirming that CN@mSiO_2_-NH_2_@Urea@PDA had been successfully prepared. The increase of 20° in the tilt angle of the hydrophobic silica sheet indicates that CN@mSiO_2_-NH_2_@Urea@PDA has excellent adhesion properties on the hydrophobic surface, which can effectively reduce the risk of being washed away by rainwater and be used as a foliar fertilizer, and the decrease of 24% in the release rate indicates that the retardation effect has been enhanced using CN@mSiO_2_-NH_2_@Urea@PDA. When replacing 40% of nitrogen fertilizer with a base fertilizer, the growth effect of Chinese cabbage was the best, and the yield of Chinese cabbage was significantly increased (69.94%). It can also significantly improve the nitrogen utilization efficiency of plants (21.64%), the nitrogen residue rate in soil layers (56.15%), and reduce the nitrogen loss rate (22.21%), while foliar spraying CN@mSiO_2_-NH_2_@Urea@PDA can also improve the nitrogen utilization efficiency of plants (18.37%), indicating that for CN@mSiO_2_-NH_2_ @Urea@PDA, the application effect was reliable. However, the safety and cost-effectiveness of the technology still require further investigation in practical large-scale applications to facilitate broader adoption and implementation. And variable weather and more soil conditions need to be taken into account under actual agricultural conditions, and this will be explored in depth in the next phase of work. In the future, the research will include a more comprehensive assessment of the toxicological properties of nanomaterials and an investigation of the degradation of nanomaterials under different soil conditions, as well as their accumulation in soil microbial communities and potential ecological impacts, to ensure their safety in agricultural applications. This study provides an effective strategy to promote the further application of core–shell nanomaterials in the agricultural field and improve farmers’ recognition of functional foliar fertilizers.

## Data Availability

The data supporting the findings of this study are available.

## Supplementary Materials

The following supporting information can be downloaded at: https://www.mdpi.com/article/10.3390/nano15070565/s1, Figure S1: TEM images of (a) CN@mSiO_2_-NH_2_, (b) CN@mSiO_2_-NH_2_@Urea and (c) CN@mSiO_2_-NH_2_@Urea@PDA; (d) The high-angle annular dark-field STEM of CN@mSiO_2_-NH2@Urea; (e) The carbon distribution in CN@mSiO_2_-NH_2_@Urea in the EDS-mapping; (f) The silicon distribution in CN@mSiO_2_-NH_2_@Urea in the EDS-mapping; (g) The oxygen distribution in CN@mSiO_2_-NH_2_@Urea in the EDS-mapping; (h) The nitrogen distribution in CN@mSiO_2_-NH_2_@Urea in the EDS-mapping; (i) EDS spectra analysis for the CN@mSiO_2_-NH_2_@Urea; Figure S2: The SEM images of CN@mSiO_2_-NH_2_@Urea after rinsing (a) 0, (b) 3, (c) 6, and (d) 9 times on silicon wafers with a magnification of 30k× times; CN@mSiO_2_-NH_2_@Urea@PAD after rinsing (e) 0, (f) 3, (g) 6, and (h) 9 times on silicon wafers with a magnification of 30k× times; Table S1: Batch combinations and processing conditions for nanocarbon preparation; Table S2: Batch combinations and processing conditions for CN@mSiO_2_ preparation; Table S3: Effect of different glucose concentration on the size of carbon nanoparticles; Table S4: Effect of different reaction time on the size of carbon nanoparticles; Table S5: Effects of different reaction temperatures on the size of carbon nanoparticles; Table S6: Effects of different TEOS concentrations on BET parameters of CN@mSiO_2_; Table S7: Effects of different ammonia concentrations on BET parameters of CN@mSiO_2_; Table S8: Influence of different ratio of alcohol to water on BET parameter of CN@mSiO_2_; Table S9: Germination indexes related to the seeds of Cauliflower heart after 5 days of incubation in different treatments; Table S10: Agronomic traits of vegetable hearts cultivated under different treatments; Table S11: Yield indicators for different treatments; Table S12: Nitrogen trend in different treated soil layers and vegetable heart.

## Abbreviations

CN	Carbon nanoparticles
mSiO_2_	Mesoporous silica dioxide
PDA	Polydopamine
DA	Dopamine
-NH_2_	Amino
SiO_2_	Silica dioxide
TEOS	Tetraethyl orthosilicate
CTAB	Cetyltriethylammnonium bromide
APTES	(3-Aminopropyl) triethoxysilane
HCl	Hydrochloric acid
C	Carbon
N	Nitrogen
O	Oxygen
Si	Silica
TEM	Transmission Electron Microscope
SEM	Scanning Electron Microscope
EDS	Energy Dispersive X-Ray Spectroscopy
FTIR	Fourier Transform Infrared Spectroscopy
XRD	X-Ray Diffraction
XPS	X-ray Photoelectron Spectroscopy
