# Supplementary material for: Preparation and Application of Core–Shell Nanocarbon-Based Slow-Release Foliar Fertilizer
Source: Nanomaterials (Basel). 2025 Apr 7;15(7):565. doi: 10.3390/nano15070565 (PMC11990319; doi:10.3390/nano15070565)
Supplement: Supplementary file 1 [file nanomaterials-15-00565-s001.zip › nanomaterials-3550337-supplementary.pdf]

Foliar slow-release dual efficacy carbon-based nanotechnology fertilizer: preparation, mechanism and application

Ting Zhang [1,2,3], Xinheng Chen [1,2,3], Hongtao Gu [1,2,3], Huayi Chen [4], Kaichun Huang [1,2,3], Jinjin Wang [1,2,3], Huijuan Xu [1,2,3], Yulong Zhang [1,2,3,*] and Wenyan Li [1,2,3,*]

[1]Guangdong Province Key Laboratory for Agricultural Resources Utilization, College of Natural Resources and Environment, South China Agricultural University, Guangzhou, 510642, China.

[2]Key Laboratory of Arable Land Conservation (South China), MOA, College of Natural Resources and Environment, South China Agricultural University, Guangzhou 510642, China.

[3]Guangdong Research Center for Agricultural Soil Pollution Prevention and Control Engineering Technology,College of Natural Resources and Environment, South China Agricultural University, Guangzhou 510642, China.

[4]School of Tropical Agriculture and Forestry, Hainan University, Haikou, 570228, China

* Correspondence: zt199812052025@163.com (T.Z.); xhchen1999@163.com (X.C.); 17633974630@163.com (H.G.); huayi93@hainanu.edu.cn (H.C.); 15863418988@163.com (K.H.); wangjinjin@scau.edu.cn (J.W.); hjxu@scau.edu.cn (H.X.); yulongzhang@scau.edu.cn (Y.Z.); lily1984191@scau.edu.cn (W.L.)

Table S1 Batch combinations and processing conditions for nanocarbon preparation

Reaction temperature /℃	Glucose concentration /(mol/L)	Reaction time /h
160	0.15	10
160	0.30	10
160	0.45	10
160	0.60	10
160	0.30	6
160	0.30	8
160	0.30	12
170	0.30	10
180	0.30	10
190	0.30	10

Table S2 Batch combinations and processing conditions for CN@mSiO_2 preparation

TEOS /(mol/L)	$NH_3 \cdot H_2O$ /(mol/L)	Alcohol-water ratio
0.006	0.18	0.6
0.011	0.18	0.6
0.017	0.18	0.6
0.023	0.18	0.6
0.017	0.11	0.6
0.017	0.15	0.6
0.017	0.21	0.6
0.017	0.18	0.4
0.017	0.18	0.8
0.017	0.18	1.0

Table S3 Effect of different glucose concentration on the size of carbon nanoparticles

Reaction temperature /℃	Glucose concentration /(mol/L)	Reaction time /h	Mean particle size /nm
160	0.15	10	71±1
160	0.30	10	96±1
160	0.45	10	170±3
160	0.60	10	162±2

Note: Data are expressed as mean ± standard error (n=5).

Table S4 Effect of different reaction time on the size of carbon nanoparticles

Reaction temperature /℃	Glucose concentration /(mol/L)	Reaction time /h	Mean particle size /nm
160	0.30	6	75±1
160	0.30	8	88±3
160	0.30	10	96±1
160	0.30	12	157±3

Note: Data are expressed as mean ± standard error (n=5).

Table S5 Effects of different reaction temperatures on the size of carbon nanoparticles

Reaction temperature /℃	Glucose concentration /(mol/L)	Reaction time /h	Mean particle size /nm
160	0.30	10	96±1
170	0.30	10	219±3
180	0.30	10	332±6
190	0.30	10	245±4

Table S6 Effects of different TEOS concentrations on BET parameters of CN@mSiO_2

TEOS (mol/L)	NH_3·H_2O (mol/L)	Alcohol-water ratio	Specific surface area (m^2/g)	Total pore volume (cm^3/g)	Aperture (nm)
0.006	0.18	0.6	121.5	0.18	1.78
0.011	0.18	0.6	302.6	0.41	1.99
0.017	0.18	0.6	481.1	0.51	2.36
0.023	0.18	0.6	552.2	0.58	2.34

Table S7 Effects of different ammonia concentrations on BET parameters of CN@mSiO_2

TEOS (mol/L)	NH_3·H_2O (mol/L)	Alcohol-water ratio	Specific surface area (m^2/g)	Total pore volume (cm^3/g)	Aperture (nm)
0.017	0.11	0.6	438.8	0.46	2.36
0.017	0.15	0.6	460.9	0.51	2.22
0.017	0.18	0.6	481.1	0.51	2.36
0.017	0.21	0.6	507.1	0.54	2.35

Table S8 Influence of different ratio of alcohol to water on BET parameter of CN@mSiO_2

TEOS (mol/L)	NH_3·H_2O (mol/L)	Alcohol-water ratio	Specific surface area (m^2/g)	Total pore volume (cm^3/g)	Aperture (nm)
0.017	0.18	0.4	386.2	0.41	1.74
0.017	0.18	0.6	481.1	0.51	2.36
0.017	0.18	0.8	478.5	0.46	2.10
0.017	0.18	1	461.1	0.52	1.97

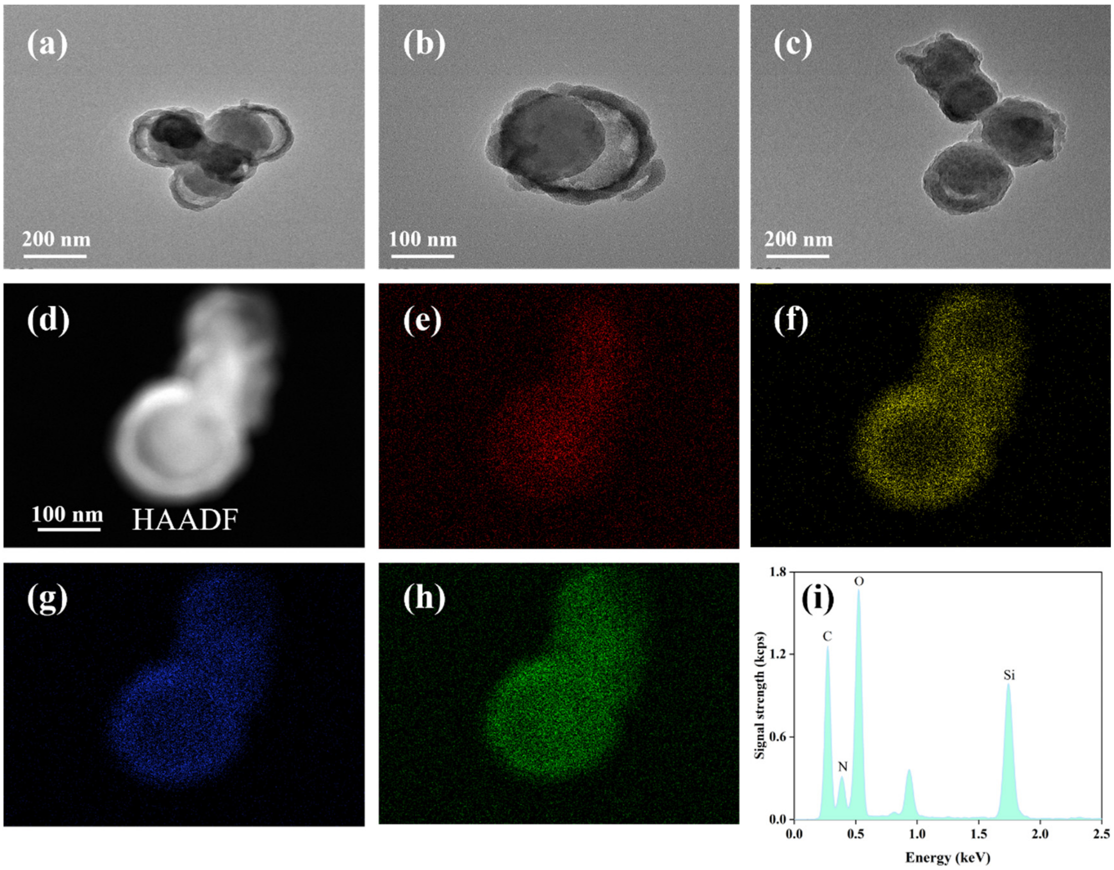

Figure S1. TEM images of (a) CN@mSiO_2-NH_2, (b) CN@mSiO_2-NH_2@Urea and (c) CN@mSiO_2-NH_2@Urea@PDA; (d) The high-angle annular dark-field STEM of CN@mSiO_2-NH_2@Urea; (e) The carbon distribution in CN@mSiO_2-NH_2@Urea in the EDS-mapping; (f) The silicon distribution in CN@mSiO_2-NH_2@Urea in the EDS-mapping; (g) The oxygen distribution in CN@mSiO_2-NH_2@Urea in the EDS-mapping; (h) The nitrogen distribution in CN@mSiO_2-NH_2@Urea in the EDS-mapping; (i) EDS spectra analysis for the CN@mSiO_2-NH_2@Urea.

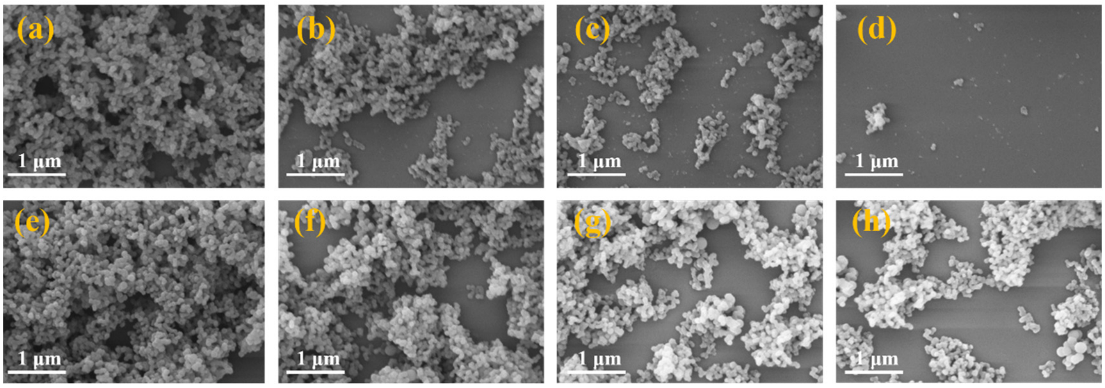

Figure S2. The SEM images of CN@mSiO_2-NH_2@Urea after rinsing (a) 0, (b) 3, (c) 6, and (d) 9 times on silicon wafers with a magnification of 30k× times; CN@mSiO_2-NH_2@Urea@PAD after rinsing (e) 0, (f) 3, (g) 6, and (h) 9 times on silicon wafers with a magnification of 30k× times.

Table S9 Germination indexes related to the seeds of Cauliflower heart after 5 days of incubation in different treatments

Treatment	Germination potential (%)	Germination rate (%)	Germination index	root length (cm)	vitality index
CK	90.00±0.00a	93.00±0.00a	61.10±0.33a	29.18±1.05b	1782±55b
T1	88.00±0.05a	93.00±0.03a	59.21±2.84a	20.18±1.58c	1186±36c
T2	93.00±0.02a	97.00±0.00a	63.72±0.76a	33.73±1.02a	2149±61a

Table S10 Agronomic traits of vegetable hearts cultivated under different treatments

Treatment	Plant height (cm)	Sedge height (cm)	Sedge rough (cm)	Blade number	SPAD
CK	7.70±0.91c	5.50±0.61d	0.42±0.01c	5.00±0.58a	45.2±4.03c
T1	19.20±0.44b	17.60±0.34c	0.59±0.03bc	5.00±0.67a	46.4±2.56c
T2	31.10±1.45a	29.00±1.78a	0.76±0.05a	6.00±0.33a	56.1±3.19ab
T3	22.60±1.85b	20.40±1.47c	0.73±0.06ab	6.00±0.33a	54.7±2.99ab
T4	20.20±1.91b	17.90±1.81c	0.56±0.02c	5.00±0.67a	52.9±0.06bc
T5	27.60±1.54a	24.70±1.22b	0.72±0.08ab	7.00±0.67a	61.6±2.03a
T6	22.40±1.15b	19.00±1.42c	0.67±0.04abc	6.00±0.33a	56.3±1.00ab

Table S11 Yield indicators for different treatments

Treatment	Fresh weight (g)	Dry weight (g)
CK	6.15±0.24e	0.47±0.04c
T1	10.05±0.52d	0.74±0.03bc
T2	17.07±1.01a	1.36±0.20a
T3	14.11±0.79bc	1.13±0.18ab
T4	8.01±0.41de	0.72±0.06bc
T5	15.78±0.99ab	1.39±0.11a
T6	12.58±1.21c	1.04±0.26ab

Table S12 Nitrogen trend in different treated soil layers and vegetable heart

Treatment	Residual nitrogen rate in soil layer (%)	Plant nitrogen use efficiency (%)	Nitrogen loss rate (%)
CK	/	/	/
T1	45.79±0.54c	6.57±0.32b	47.63±0.83ab
T2	56.15±0.29a	21.64±3.85a	22.21±3.99d
T3	51.32±0.62b	15.35±1.33ab	33.32±1.91cd
T4	41.81±0.94d	6.63±1.07b	51.56±1.50a
T5	44.54±1.76cd	18.37±1.73a	37.09±3.45bc
T6	43.16±1.84cd	13.36±4.97ab	43.48±6.78abc
